# Autism Spectrum Disorder and Intellectual Disability: A Pilot Study Exploring Associations between Child Sleep Problems, Child Factors and Parent Factors

**DOI:** 10.3390/ijerph182111377

**Published:** 2021-10-29

**Authors:** Nicole Papadopoulos, Chloe Emonson, Christina Martin, Emma Sciberras, Harriet Hiscock, Samantha Lewis, Jane McGillivray, Nicole Rinehart

**Affiliations:** 1 Deakin Child Study Centre, School of Psychology, Faculty of Health, Deakin University, Geelong, VIC 3220, Australia; chloe.emonson@deakin.edu.au (C.E.); machri@deakin.edu.au (C.M.); emma.sciberras@deakin.edu.au (E.S.); s.lewis@deakin.edu.au (S.L.); jane.mcgillivray@deakin.edu.au (J.M.); nicole.rinehart@monash.edu (N.R.); 2 Murdoch Children’s Research Institute, Parkville, VIC 3052, Australia; harriet.hiscock@rch.org.au; 3 Department of Paediatrics, University of Melbourne, Parkville, VIC 3052, Australia; 4 The Royal Children’s Hospital, Parkville, VIC 3052, Australia; 5 School of Educational Psychology and Counselling, Faculty of Education, Monash University, Clayton, VIC 3800, Australia

**Keywords:** autism spectrum disorder, intellectual disability, sleep problems, children, parents

## Abstract

Sleep problems are common in children with autism spectrum disorder (ASD). However, few studies have undertaken group comparisons of sleep profiles and factors associated with poorer sleep between children with ASD without intellectual disability (ID; hereafter referred to as ASD) and ASD with co-occurring ID (hereafter referred to as ASD + ID). This study aimed to (1) compare child (sleep problems and emotional and behavioural problems (EBPs)) and parent factors (parenting stress and mental health) for children with ASD compared to children with ASD + ID, and (2) examine the associations between sleep problems and child and parent factors in both groups. Parents of 56 children with ASD (22 ASD, 34 ASD + ID) aged 6–13 years took part in the study. No statistically significant differences in sleep problems were found between children with ASD compared to children with ASD + ID. However, total EBPs were independently associated with child sleep problems in both groups. Further, ‘Self-Absorbed’ and ‘Communication Disturbance’ EBPs were significantly greater in the ASD + ID compared to the ASD group. Overall treatment outcomes for children with ASD may be further improved if consideration is given to the specific types of EBPs being experienced by the child and their association with sleep problems.

## 1. Introduction

Autism spectrum disorder (ASD) is a neurodevelopmental disorder characterised by persistent social communication and interaction difficulties, and restricted, repetitive behaviours or interests [1]. ASD affects approximately 1.5–2.5% of Australian children [2], and approximately 31–70% have a co-occurring intellectual disability (ID) [3,4]. Children who meet the Diagnostic and Statistical Manual of Mental Disorders—Fifth Edition (DSM-5) criteria for ID have impairments in cognitive functioning and difficulties with adaptive functioning [1]. Many of the symptoms of both ASD and ID overlap, creating a challenge for the nosology and diagnosis of ASD or ID alone or as co-occurring conditions [3]. Other co-occurring conditions are common for children with ASD, with approximately 62–79% also experiencing emotional and behavioural problems [5,6].

Approximately 50–80% of children with ASD experience sleep problems [7,8,9]. Common sleep problems include long sleep-onset latency (time to fall asleep), less overall time asleep, increased overnight awakenings, and poor sleep efficiency (i.e., total sleep time versus total time in bed; [9]). Sleep problems in children with ASD can persist over time [10] and be present across the lifespan [11,12]. There are a multitude of factors that contribute to sleep problems in children with ASD; for example, biological factors (e.g., melatonin rhythm abnormalities), behavioural factors (e.g., repetition or routine difficulties), and the presence of co-occurring psychiatric conditions (e.g., attention-deficit/hyperactivity disorder (ADHD) and anxiety; [13]), with behavioural origins being most common [14,15].

The role of intelligence quotient (IQ) in understanding sleep problems is important to consider, as approximately 76% of children with an idiopathic diagnosis of ID, who may have co-occurring psychiatric (e.g., anxiety) and medical conditions (e.g., epilepsy) but do not have ASD, experience sleep problems [16]. For this group, the two most common sleep problems experienced include bedtime resistance and sleep anxiety [16]. Although many studies have examined the influence of developmental level or IQ on the sleep profiles of children with ASD (e.g., [17,18,19,20,21]), few studies have specifically undertaken group comparisons between children with ASD but not ID and ASD with co-occurring ID (ASD + ID; e.g., [22]).

A meta-analysis by Elrod and Hood [22] found that co-occurring ID significantly moderated the relationship between ASD and children’s total sleep time as measured by actigraphy or polysomnography. Results showed that only studies that included children with ASD + ID found significantly less total sleep time in children with ASD than their typically developing peers, whereas studies that excluded children with ID did not show a significant difference [22]. However, a study by Hollway and Aman [23] found that IQ was not a significant predictor of subjectively measured overall sleep problems in children with ASD (*n* = 1583); instead, they incorporated level of intellectual functioning as a vulnerability factor in their bidirectional theoretical framework for insomnia in children with ASDs. This framework positions poor reciprocal social interactions (an ASD symptom), gastrointestinal problems, and internalising symptoms as more directly impairing sleep in children with ASD [23].

ASD symptom severity and emotional and behavioural problems are consistently identified as correlates of sleep problems in paediatric populations [24,25]. One study assessed emotional and behavioural problems in 277 young children with ASD (aged 4–8 years) with and without ID, reporting that children with ASD and an IQ ≥ 70 exhibited higher scores for disruptive behaviour, depression and anxiety symptoms, while children with ASD and IQ < 70 exhibited higher self-absorbed and hyperactivity scores [6]. However, this study did not consider sleep. Given the prevalence of emotional and behavioural problems experienced by children with ASD [6] and the association with sleep problems [24,25], further research is needed to understand potential differences in the types of emotional and behavioural problems experienced and their associations with sleep difficulties for children with ASD without ID versus ASD + ID.

Parent factors, such as mental health difficulties and elevated stress levels, have also been proposed as factors associated with sleep problems for children with ASD [26,27]. A systematic review of the relationship between family functioning and child sleep problems in ADHD and ASD by Martin et al. [26] identified an association between child sleep problems and parenting stress and parent mental health in families with children with ASD. However, only three of the included studies measured intellectual functioning and none compared the associations for children with ASD + ID versus ASD without ID [26].

In summary, behavioural sleep disorders are prevalent in ASD and likely underpinned by a multitude of risk and protective factors. Many children with ASD will have ID; however, the majority of the ASD sleep literature is focused on children without ID or reports on mixed samples of children with ASD (i.e., have not separately examined children with and without ID). Few studies have undertaken group comparisons between children with ASD without ID and ASD + ID in relation to sleep problems and emotional and behavioural problems ( e.g., [6,22]). In addition, to the best of our knowledge, no studies have conducted group comparisons related to parent factors (i.e., parenting stress and parent mental health) or for sleep problems using a measure of sleep specifically designed for children with ASD [28]. An understanding of the types of sleep problems that children with ASD without ID versus ASD + ID experience, along with an understanding of potential differences in associations between sleep problems and child and parent factors among the two groups, is important to inform interventions for children with ASD who have complex needs.

Therefore, the aims of this study were to: (1) compare child factors (sleep problems, and emotional and behavioural problems) and parent factors (parenting stress and parent mental health) between two groups of children, children with ASD + ID and children with ASD without ID (hereafter referred to as the ‘ASD’ group); and (2) investigate the independent associations between child sleep problems and each of: (i) child emotional and behavioural problems, (ii) ASD symptom severity, (iii) parenting stress, and (iv) parent mental health in both groups. Regarding our first aim, we hypothesise that there will be a difference in the sleep problems experienced by children with ASD and children with ASD + ID. We also hypothesise that children with ASD + ID will experience greater emotional and behavioural problems than children with ASD. For our second aim, we hypothesise that independent positive associations will exist between sleep problems experienced by children with ASD and ASD + ID and each of the following health outcomes: (i) child emotional and behavioural problems, (ii) ASD symptom severity, (iii) parenting stress and (iv) parent mental health difficulties.

## 2. Materials and Methods

### 2.1. Participants

Participants were 56 children with ASD (34 ASD + ID, 22 ASD) aged 6–13 years (*M* = 9.18, *SD* = 2.23) and their parents (*M_age_* = 41.57 years, *SD* = 5.87). The study was advertised Australia-wide in paediatric, occupational therapist, and psychologist clinics, and autism support and early intervention services. The study was also advertised through ASD support groups on social media. Families were included if their child had a diagnosis of ASD according to the DSM criteria, confirmed by parents providing a clinical report containing a formal diagnosis. Clinical reports were also used to confirm ID diagnosis if relevant. Children with ASD or ASD + ID who had parent-reported co-occurring ADHD were also included. We acknowledge that although we have categorised our sample into two groups (ASD and ASD + ID) for research purposes in accordance with previous research studies (e.g., [6,22]), this does not take into consideration the complexity and heterogeneity of symptom severity within groups.

### 2.2. Measures

#### 2.2.1. Child Sleep Problems

Child sleep problems were measured by parent-report using the ASD scoring for the Children’s Sleep Habits Questionnaire (CSHQ-ASD) proposed by Katz et al. [28], and previously used with families of children with ASD [27,29,30]. The total score was used (23 items), as were all four subscales: Sleep Initiation and Duration (e.g., Child sleeps the right amount), Sleep Anxiety/Co-Sleeping (e.g., Needs parent in the room to fall asleep), Night Waking/Parasomnias (e.g., Awakens alarmed by a frightening dream) and Daytime Alertness (e.g., Has difficulty getting out of bed in the morning). Each item was rated using a 3-point scale from 1 = rarely to 3 = usually, indicating the frequency of the sleep problem over the past week. Some items were reverse-coded, with higher scores indicating more sleep problems. When using a clinical sample, the Sleep Initiation and Duration, Sleep Anxiety/Co-Sleeping and Daytime Alertness subscales demonstrated acceptable internal consistency (α = 0.80 − 0.81), while the internal consistency coefficient for Night Waking/Parasomnias was 0.67 [28].

#### 2.2.2. Child Emotional and Behavioural Problems

Child emotional and behavioural problems were measured using the Developmental Behaviour Checklist Primary Carer version (DBC-P) [31]. This measure is considered reliable and valid for use with children with ID, with high test-retest reliability (Intraclass Correlation (ICC) = 0.83), internal consistency (α = 0.94), and inter-rater reliability for parents (ICC = 0.80) [31]. This measure has also been used with families of children with ASD [6]. The DBC-P total score was used, as were each of its subscales: Disruptive (e.g., Kicks, hits others), Antisocial (e.g., Tells lies), Self-Absorbed (e.g., Eats non-food items e.g., dirt, grass, soap), Communication Disturbance (e.g., Talks to self or imaginary people or objects), Anxiety (e.g., Distressed about being alone) and Social Relating (e.g., Doesn’t show affection). Each item was rated on a three-point scale from 0 = not true as far as you know to 2 = very true or often true, indicating whether the behaviour was present in the previous six months. Higher scores indicate more emotional and behavioural problems. 

#### 2.2.3. Parenting Stress

Parenting stress was measured by parent self-report using the Parenting Stress Index —Short Form 4th Edition (PSI-SF-4) [32]. The total score (36 items) and three subscales were used: Parental Distress (e.g., I often have the feeling that I cannot handle things very well), Parent-Child Dysfunctional Interactions (e.g., My child smiles at me much less than I expected) and Difficult Child (e.g., My child seems to cry or fuss more often than most children). Each item is rated on a five-point scale from 1 *=* Strongly Disagree to 5 = Strongly Agree, with higher scores indicating increased stress. The Defensive Responding validity scale was also used, with scores of ≤10 being indicative of parents potentially engaging in biased responding. The PSI-SF is reported to have high internal consistency (α = 0.91) when used with children with ASD [33], and the PSI-SF-4 has been used previously with families of children with ASD [30,34].

#### 2.2.4. Parent Mental Health

Parent mental health was measured by parent self-report using the Kessler Psychological Distress Scale (K10) [35], which has been used previously with families of children with ASD [36]. The total score, comprising 10 items, was used. Each item is rated on a five-point scale from 1 = none of the time to 5 = all of the time, indicating the frequency of the item over the past 30 days. Higher scores indicate more psychological distress. The K10 demonstrates consistent psychometric properties and can discriminate between clinical and non-clinical presentations [35].

#### 2.2.5. Sample Characteristics

Parents were asked to report demographic information about themselves and their child. Child information included sex, age, whether the child had a co-occurring ADHD diagnosis, and ASD symptom severity measured using the total score on the Social Communication Questionnaire Lifetime form [37]. Parent information included sex, date of birth (which was converted to age in years based on the date of survey completion), living arrangements (from the following options: child lives with mother and father, mostly with mother, mostly with father, or other), the parent’s level of education, their employment status, and their relationship status (using the following selections: married, de facto/living together, divorced, separated, never married, or widowed).

### 2.3. Procedure

The Deakin University Human Research Ethics Committee approved this study (2016-080). Parents registered their interest in participating in the study via the study website or by contacting the research team directly. A member of the research team screened individuals via phone, which required parents to confirm their child was aged between 6–13 years and had a diagnosis of ASD or ASD + ID. A member of the research team confirmed eligibility by sighting the child’s diagnosis in the relevant clinical reports. Eligible families were sent an individual link to the plain language statement, consent form, and online survey. Informed consent was required prior to beginning the survey. At the conclusion of the study, each family was mailed a supermarket gift voucher.

### 2.4. Data Analysis

Sample characteristics and study variables were reported, using mean (*M*) and standard deviations (*SD*) for continuous variables and percentages for categorical variables. Sample size was considered sufficient for a pilot study [38]. For aim 1, independent sample *t*-tests were undertaken to investigate differences between the ASD and ASD + ID groups for our study variables (i.e., sleep problems, emotional and behavioural problems, parenting stress and parent mental health), with Cohen’s *d* effect sizes also reported [39]. Bootstrapping was undertaken when independent sample *t*-test assumptions were violated. For aim 2, linear regression analyses were undertaken to examine independent associations between child and parent factors (i.e., ASD symptom severity, emotional and behavioural problems, parenting stress, and parent mental health) and total sleep problems for each group separately. Both unadjusted and adjusted linear regression analyses were undertaken. In adjusted analyses, all child and parent factors were entered as independent variables to examine their unique contribution to sleep problems. Assumptions required to undertake regression analyses were not violated and effect sizes were interpreted in line with Ferguson [40]. All analyses were conducted using Stata 15.0 [41].

## 3. Results

### 3.1. Sample Characteristics

Sample characteristics are detailed in Table 1. The majority of children were male (ASD + ID: 91%; ASD: 73%) and lived with both parents (ASD + ID: 77%; ASD: 86%). Approximately one-third of children had a co-occurring diagnosis of ADHD (36%). The ADHD prevalence rates differed between groups (ASD + ID: 29%; ASD: 46%). Parent respondents were primarily female (ASD + ID: 91%; ASD: 82%). Nearly half the parents had completed tertiary study (ASD + ID: 41%; ASD: 50%), and more than half were employed (ASD + ID: 68%; ASD: 55%) and in a relationship (ASD + ID: 79%; ASD: 86%).

### 3.2. Group Comparisons

Group comparisons for child sleep problems and child emotional and behavioural problems are detailed in Table 2. There were no significant differences between children in the ASD and ASD + ID groups for total sleep problems or the individual sleep problem domains. However, medium-to-large effect sizes were evident for total sleep problems, Night Waking/Parasomnias and Daytime Alertness, with the ASD group being higher in each case. Medium-to-large effect sizes were also evident for Sleep Initiation and Duration where the ASD + ID group was higher, indicating that this group may experience more difficulty with sleep onset and insufficient sleep. There were no significant differences for total emotional and behavioural problems between children in the ASD and ASD + ID groups. However, differences did exist when comparing specific emotional and behavioural problem domains. Children in the ASD + ID group experienced significantly higher levels of Self-Absorption (*M* = 18.38, *SD* = 7.89) and Communication Disturbance (*M* = 6.44, *SD* = 3.88) than children in the ASD group (*M* = 13.05, *SD* = 7.44; *M* = 4.50, *SD* = 3.05), with medium-to-large effect.

Group comparisons for parenting stress and parent mental health are outlined in Table 3. There were no significant differences between parents of children in the ASD and ASD + ID groups for parent mental health, total parenting stress, or the individual parenting stress domains. However, small-to-medium effects were evident for total parenting stress and Difficult Child, and medium-to-large effects were evident for Parent–Child Dysfunctional Interaction, with the ASD + ID group being higher in each case, indicating that this group may experience increased stress.

### 3.3. Associations between Child Factors, Parent Factors and Sleep Problems

Unadjusted associations between child factors, parent factors and total sleep problems for the ASD + ID and ASD groups are detailed in Table 4. For the ASD + ID group, total emotional and behavioural problems (*β =* 0.55) had a significant association with total sleep problems, with moderate effect. For the ASD group, total emotional and behavioural problems (*β =* 0.76) and parent mental health (*β =* 0.48) were significantly associated with total sleep problems, with moderate and small effect, respectively.

Independent associations between child factors (ASD symptom severity and emotional and behavioural problems), parent factors (parent mental health and parenting stress) and total sleep problems are reported in Table 5. For the ASD + ID group, total emotional and behavioural problems were independently associated with total sleep problems (*β =* 0.54, *p* = 0.005). For the ASD group, total emotional and behavioural problems were independently associated with total sleep problems (*β =* 0.81, *p* = 0.002).

## 4. Discussion

A core aim of this paper was to investigate the differences in sleep problems and the factors associated with sleep problems for children with ASD and children with more complex needs, defined here as ASD + ID. The study found no statistically significant group differences in the severity of total or specific sleep problems experienced by children with ASD versus children with ASD + ID. Additionally, no significant differences were found between the groups on parenting stress or parent mental health. ASD symptom severity, parenting stress and parent mental health were not found to be independently associated with child sleep problems in either group in this study. However, total emotional and behavioural problems were associated with child sleep problems for both groups. Further, significant differences in the specific types of emotional and behavioural problems experienced by children in the two groups were identified.

Whilst we found no statistically significant differences in the severity of sleep problems between the two groups, small-to-medium effect sizes were detected between groups for several types of sleep problems. In particular, children with ASD demonstrated more difficulties with Night Waking/Parasomnias, Daytime Alertness and total sleep problems, whereas children with ASD + ID demonstrated more difficulty with Sleep Initiation and Duration with a medium-effect size, which supports the meta-analysis findings by Elrood and Hood [22], who reported shorter sleep duration for children with ASD + ID. These results suggest that ID may exacerbate some but not all types of sleep problems experienced by children with ASD.

There were significant associations between total emotional and behavioural problems and sleep problems in both groups. Notably, these associations held when accounting for other important clinical factors explored in this study. Previous studies investigating sleep problems in children with ASD have not controlled for children’s emotional and behavioural problems (e.g., [6,25,42,43]). This finding highlights the importance of controlling for these factors when investigating sleep problems in children with ASD, in order to better understand the sleep profile and to guide clinical practice.

Additionally, in comparing specific types of emotional and behavioural problems between the two groups, results revealed significant differences on two individual emotional and behavioural subscales. Children with ASD + ID had significantly increased levels of Self-Absorbed Behaviour and Communication Disturbance compared to children with ASD, with medium-to-large effect. This aligns with previous research by Chandler et al. [6], who found that children with ASD with IQ < 70 experience higher rates of self-absorption than children with ASD with IQ > 70. It can be challenging to understand whether the greater difficulties with communication and self-absorbed behaviour evident in children with ASD + ID are a result of their cognitive difficulties or severity of ASD symptomatology [44]. However, it is important that clinicians are aware of the potential association between emotional and behavioural problems and sleep problems for all children with ASD and the specific emotional and behavioural problems that children may be experiencing, in order to appropriately tailor interventions to best suit the needs of the child.

Notably, the results of this study did not demonstrate a significant association between sleep problems and ASD symptom severity in either the ASD or ASD + ID group. This is in contrast to previous literature. For example, Tudor et al. [43] found associations between children’s sleep problems and three autism symptom domains (stereotyped behaviour, communication, and social interaction) and overall autism severity. Similarly, Hollway et al. [23] found an association between sleep disturbance and social and communication symptom severity but not restricted and repetitive behaviour severity. Mazurek and Petroski [45] found a positive relationship between the severity of children’s sleep problems and sensory over responsivity. Further, Adams et al. [42] found relationships between sleep problems and ASD symptom severity that may be bidirectional [42]. It is possible that sample and methodological differences contribute to the inconsistency. For example, all studies had larger samples compared to the current study. The study by Tudor et al. [43] included children with autism only (i.e., without ID or other co-occurring developmental disability diagnoses). ASD diagnosis was not required in the Adams et al. [42] study, and co-occurring ID was not reported. Finally, unlike the current study, participants were not analysed according to ID in the Hollway et al. [23] or Mazurek and Petroski [45] studies.

Our results showed no significant differences between groups for total parenting stress, the individual parenting stress subscales, or parent mental health. However, a medium-to-large effect was observed for the Parent–Child Dysfunctional Interactions subscale and a small-to-medium effect was observed for the Difficult Child subscale and total parenting stress scores. This suggests that this study may be underpowered to identify statistically significant differences, but that there may be differences between the groups that are important to consider clinically. There is limited literature explicitly comparing parent mental health between parents of children with ASD with and without ID [46], as most studies analysed a mixed sample (i.e., ASD and ASD + ID combined, e.g., [47]). The use of mixed samples can be a limitation due to heterogeneity of intellectual ability [48]. Further, it is possible that child factors such as emotional and behavioural problems, which appear to differ between children with ASD and ASD + ID [6], influence the levels of parenting stress experienced. In support of this emerging hypothesis, emotional and behavioural problems have been found to contribute to one-third of maternal stress in mothers of children with ASD + ID [49]. Further research should therefore be conducted with larger samples to explore differences in stress and mental health of parents of children with ASD and ASD + ID.

We did not find a significant independent association between children’s sleep problems and parenting stress or children’s sleep problems and parent mental health in either group. However, a small significant association was evident between sleep problems and parent mental health in the ASD group before controlling for other child factors (i.e., ASD symptom severity, emotional and behavioural problems) or parenting stress. This is consistent with prior research that did not control for any additional factors [50] or controlled for ASD symptom severity only [51].

This study had several strengths, including confirmation of the clinical diagnosis of ASD and ID for each child, inclusion of children with ASD with and without ID, and use of validated measures of sleep difficulties, ASD symptom severity, emotional and behavioural problems, parenting stress, and parent mental health. In addition, sleep difficulties were assessed using a measure designed specifically for children with ASD [28]. Despite this, some limitations should be considered when interpreting the findings. Firstly, despite our ASD + ID sample being larger than some other studies, the sample size is small, which likely impacted the power of the study. Our findings should therefore be considered preliminary. Additionally, although clinical assessment reports were used to confirm participant diagnoses of ASD and ID (where applicable), a cognitive measure was not employed and ID was analysed as a categorical variable (i.e., present or not present). This meant that we were unable to explore the influence of mild, moderate or severe levels of intellectual functioning or to describe the heterogeneity of our clinical sample. This study also relied on subjective parent-report measures to assess sleep problems, which may impact accuracy. Future research could supplement subjective sleep measures with objective measures such as actigraphy [52]. However, sensory sensitivities in autism may preclude the successful use of such objective measures (e.g., see [53]).

The preliminary findings of this study have potential clinical implications. Firstly, the relationship between overall emotional and behavioural problems and sleep difficulties for all children in this sample and the identified group differences in some aspects of emotional and behavioural problems highlight the importance of developing a nuanced assessment of each individual child, and tailoring interventions and treatment plans to account for the diverse presentations of children with ASD. For example, if a child experiences emotional or behavioural problems, it may be useful to discuss the sequencing of interventions with the family to determine the preferred approach (i.e., targeting emotional/behavioural or sleep difficulties first). Secondly, behavioural sleep interventions that have been shown to be successful in treating behavioural sleep problems in children with ASD without ID [54,55] may also be helpful in treating the same sleep problems in children with ASD + ID. In addition, given the emotional and behavioural problems experienced by children with ASD and the possible mental health difficulties and stress experienced by their parents, it may also be useful for clinicians to consider the family unit in planning treatment, to determine how they can best facilitate parents’ implementation of sleep intervention strategies in the home environment.

## 5. Conclusions

Although 31–70% of children with ASD have an ID, little is known about child and parent factors that may contribute to behavioural sleep difficulties in children with ASD + ID. The findings of the current study provide preliminary evidence that children’s emotional and behavioural problems are associated with sleep difficulties for children with ASD with and without ID, and that some emotional and behavioural problems experienced by children with ASD + ID may differ compared to children with ASD without ID. Further research is needed to better understand this difference in order to inform potential adaptations to existing behavioural sleep interventions currently used to treat sleep problems in children with ASD without ID.

## Figures and Tables

**Table 1:** Sample Characteristics of Parents and Children with ASD and ASD + ID.

Variable	ASD + ID (*n* = 34)	ASD (*n* = 22)	*p*
Child			
Male gender, *n* (%)	31 (91.2)	16 (72.7)	0.133
Age in years, *M* (*SD*)	8.91 (2.21)	9.59 (2.26)	0.270
ASD symptom severity, *M (SD)*	25.41 (4.88)	23.41 (5.73)	0.167
Co-occurring ADHD, *n* (%)	10 (29.4)	10 (45.5)	0.262
Lives with both parents, *n* (%)	26 (76.5)	19 (86.4)	0.679
Parent			
Female gender, *n* (%)	31 (91.2)	18 (81.8)	0.415
Age in years, *M* (*SD*)	41.03 (5.90)	42.41 (5.86)	0.395
Education, *n* (%)			0.610
Completed Year 10 only	3 (8.8)	2 (9.1)	
Completed high school only	3 (8.8)	0 (0.0)	
Completed tertiary study	14 (41.2)	11 (50.0)	
Relationship status, *n* (%)			0.060
Couple	27 (79.4)	19 (86.4)	
Single	7 (20.6)	3 (13.6)	
Employed, *n* (%)	23 (67.6)	12 (54.5)	0.322

Note: ADHD = Attention deficit hyperactivity disorder. ASD = Autism Spectrum Disorder. ID = Intellectual disability.

**Table 2:** Group Comparisons of Child Sleep Problems and Emotional and Behavioural Problems.

	ASD + ID	ASD			
Variable	M (SD) (*n* = 34)	M (SD) (*n* = 22)	*t*	*p*	*d*
Sleep Problems (parent-reported)					
Sleep Initiation and Duration	13.12 (1.63)	12.45 (1.30)	1.61	0.114	0.45
Sleep Anxiety/Co-Sleeping	8.21 (1.43)	8.45 (1.44)	−0.63	0.521	0.17
Night Waking/Parasomnias	9.09 (2.29)	10.09 (1.95)	−1.69	0.131	0.47
Daytime Alertness	9.91 (2.75)	10.91 (3.01)	−1.28	0.224	0.35
Sleep Problems Total	40.32 (4.23)	41.91 (4.45)	−1.34	0.185	0.37
Emotional & Behavioural Problems (parent-reported)					
Disruptive	18.85 (8.50)	16.32 (9.22)	1.05	0.296	0.29
Antisocial	1.62 (1.58)	2.36 (2.08)	−1.52	0.167	0.55
Self-Absorbed	18.38 (7.89)	13.05 (7.44)	2.53	0.015	0.70
Communication Disturbance	6.44 (3.88)	4.50 (3.05)	1.98	0.029	0.56
Anxiety	12.26 (4.80)	11.82 (5.03)	0.33	0.740	0.09
Social Relating	8.91 (3.83)	7.18 (3.30)	1.74	0.088	0.48
Emotional & Behavioural Problems Total	77.85 (23.60)	65.00 (25.36)	1.93	0.058	0.53

**Table 3:** Group Comparisons of Parenting Stress and Parent Mental Health.

	ASD + ID	ASD			
Variable	M (SD) (*n* = 34)	M (SD) (*n* = 22)	*t*	*p*	*d*
Parenting Stress (self-reported)					
Parental Distress	38.06 (9.96)	39.40 (7.52)	−0.52	0.605	0.15
Parent-Child Dysfunctional Interaction	35.26 (8.58)	30.75 (7.45)	1.96	0.056	0.56
Difficult Child	43.97 (8.31)	40.15 (8.65)	1.61	0.114	0.45
Total Parenting Stress	117.29 (22.21)	110.30 (17.40)	1.21	0.233	0.35
Total Parent Mental Health (self-reported)	23.76 (6.95)	22.59 (7.40)	0.60	0.550	0.16

**Table 4:** Unadjusted Associations between Child Factors, Parent Factors and Child Sleep Problems.

Variable	Sleep Problems							
	ASD + ID ^a^				ASD ^b^			
	β	95% CI	*p*	R^2^	β	95% CI	*p*	R^2^
ASD Symptom Severity	0.11	[−0.22, 0.40]	0.549	0.01	−0.16	[−0.48, 0.23]	0.479	0.03
Emotional & Behavioural Problems	0.55	[0.05, 0.15]	0.001	0.31	0.76	[0.08, 0.19]	<0.001	0.58
Parent Mental Health	0.27	[−0.05, 0.38]	0.121	0.07	0.48	[0.04, 0.53]	0.025	0.23
Parenting Stress	0.30	[−0.01, 0.12]	0.084	0.09	0.34	[−0.03, 0.20]	0.138	0.12

Note: ^a^ *n* = 34 ^b^ *n* = 20–22.

**Table 5:** Independent Associations between Child Factors, Parent Factors and Child Sleep Problems.

Variable	Sleep Problems					
	ASD + ID ^a^			ASD ^b^		
	β	95% CI	*p*	β	95% CI	*p*
ASD Symptom Severity	−0.18	[−0.47, 0.16]	0.334	−0.06	[−0.34, 0.25]	0.743
Emotional & Behavioural Problems	0.54	[0.03, 0.16]	0.005	0.81	[0.06, 0.22]	0.002
Parent Mental Health	0.05	[−0.19, 0.25]	0.775	0.12	[−0.21, 0.35]	0.060
Parenting Stress	0.13	[−0.05, 0.10]	0.516	−0.15	[−0.13, 0.07]	0.520

Note: ^a^ *n* = 34. ^b^ *n* = 22.

## Data Availability

Data from this study has not been made publicly available.

## References

[1] American Psychiatric Association (2013). Diagnostic and Statistical Manual of Mental Disorders.

[2] Randall M., Sciberras E., Brignell A., Ihsen E., Efron D., Dissanayake C., Williams K. (2015). Autism spectrum disorder: Presentation and prevalence in a nationally representative Australian sample. Aust. N. Z. J. Psychiatry.

[3] Matson J.L., Shoemaker M. (2009). Intellectual disability and its relationship to autism spectrum disorders. Res. Dev. Disabil..

[4] Baio J., Wiggins L., Christensen D.L., Maenner M.J., Daniels J., Warren Z. (2018). Prevalence of autism spectrum disorder among children aged 8 years autism and developmental disabilities monitoring network, 11 sites, United States, 2014. Morb. Mortal. Wkly. Rep. Surveill. Summ..

[5] Simonoff E., Pickles A., Charman T., Chandler S., Loucas T., Baird G. (2008). Psychiatric disorders in children with autism spectrum disorders: Prevalence, comorbidity, and associated factors in a population-derived sample. J. Am. Acad. Child Adolesc. Psychiatry.

[6] Chandler S., Howlin P., Simonoff E., O’Sullivan T., Tseng E., Kennedy J., Charman T., Baird G. (2015). Emotional and behavioural problems in young children with autism spectrum disorder. Dev. Med. Child Neurol..

[7] Richdale A.L., Schreck K.A. (2009). Sleep problems in autism spectrum disorders: Prevalence, nature, & possible biopsychosocial aetiologies. Sleep Med. Rev..

[8] Reynolds A.M., Malow B.A. (2011). Sleep and autism spectrum disorders. Pediatr. Clin. N. Am..

[9] Cohen S., Conduit R., Lockley S.W., Rajaratnam S.M., Cornish K.M. (2014). The relationship between sleep and behavior in autism spectrum disorder (ASD): A review. J. Neurodev. Disord..

[10] May T., Cornish K., Conduit R., Rajaratnam S., Rinehart N. (2015). Sleep in high-functioning children with autism: Longitudinal developmental change and associations with behavior problems. Behav. Sleep Med..

[11] Cortesi F., Giannotti F., Ivanenko A., Johnson K. (2010). Sleep in children with autistic spectrum disorder. Sleep Med..

[12] Baker E.K., Richdale A.L. (2017). Examining the behavioural sleep-wake rhythm in adults with autism spectrum disorder and no comorbid intellectual disability. J. Autism Dev. Disord..

[13] Reynolds A.M., Soke G.N., Sabourin K.R., Hepburn S., Katz T., Wiggins L.D., Schieve L.A., Levy S.E. (2019). Sleep problems in 2- to 5-year-olds with autism spectrum disorder and other developmental delays. Pediatrics.

[14] Souders M.C., Mason T.B.A., Valladares O., Levy S.E., Mandell D.S., Weaver T.E., Pinto-Martin J., Bucan M. (2009). Sleep behaviors and sleep quality in children with autism spectrum disorders. Sleep.

[15] Malow B.A., McGrew S.G. (2008). Sleep disturbances and autism. Sleep Med. Clin..

[16] Köse S., Yılmaz H., Ocakoğlu F.T., Özbaran N.B. (2017). Sleep problems in children with autism spectrum disorder and intellectual disability without autism spectrum disorder. Sleep Med..

[17] Hoshino Y., Watanabe H., Yashima Y., Kaneko M., Kumashiro H. (1984). An investigation on sleep disturbance of autistic children. Psychiatry Clin. Neurosci..

[18] Patzold L., Richdale A., Tonge B. (1998). An investigation into sleep characteristics of children with autism and Asperger’s Disorder. J. Paediatr. Child Heal..

[19] Bradley E.A., Summers J.A., Wood H.L., Bryson S.E. (2004). Comparing rates of psychiatric and behavior disorders in adolescents and young adults with severe intellectual disability with and without autism. J. Autism Dev. Disord..

[20] Taylor M.A., Schreck K.A., Mulick J.A. (2012). Sleep disruption as a correlate to cognitive and adaptive behavior problems in autism spectrum disorders. Res. Dev. Disabil..

[21] Cohen S., Fulcher B.D., Rajaratnam S., Conduit R., Sullivan J.P., Hilaire M.A.S., Phillips A.J., Loddenkemper T., Kothare S.V., McConnell K. (2017). Behaviorally-determined sleep phenotypes are robustly associated with adaptive functioning in individuals with low functioning autism. Sci. Rep..

[22] Elrod M.G., Hood B.S. (2015). Sleep differences among children with autism spectrum disorders and typically developing peers: A meta-analysis. J. Dev. Behav. Pediatrics.

[23] Hollway J.A., Aman M.G., Butter E. (2013). Correlates and Risk Markers for Sleep Disturbance in Participants of the Autism Treatment Network. J. Autism Dev. Disord..

[24] Hollway J.A., Aman M.G. (2011). Sleep correlates of pervasive developmental disorders: A review of the literature. Res. Dev. Disabil..

[25] Lindor E., Sivaratnam C., May T., Stefanac N., Howells K., Rinehart N. (2019). Problem behavior in autism spectrum disorder: Considering core symptom severity and accompanying sleep disturbance. Front. Psychiatry.

[26] Martin C.A., Papadopoulos N., Chellew T., Rinehart N.J., Sciberras E. (2019). Associations between parenting stress, parent mental health and child sleep problems for children with ADHD and ASD: Systematic review. Res. Dev. Disabil..

[27] Martin C.A., Sciberras E., Papadopoulos N., Engel L., Hiscock H., Williams K., Howlin P., McGillivray J., Rinehart N.J. (2021). Associations between child sleep problem severity and maternal well-being in children with autism spectrum disorder. J. Autism Dev. Disord..

[28] Katz T., Shui A.M., Johnson C.R., Richdale A.L., Reynolds A.M., Scahill L., Malow B.A. (2018). Modification of the Children’s Sleep Habits Questionnaire for Children with Autism Spectrum Disorder. J. Autism Dev. Disord..

[29] MacDonald L.L., Gray L., Loring W., Wyatt A., Bonnet K., Schlund D., Gaston M.L., Malow B.A. (2021). A community-based sleep educational intervention for children with autism spectrum disorder. Res. Autism Spectr. Disord..

[30] Johnson C.R., Smith T., DeMand A., Lecavalier L., Evans V., Gurka M., Swiezy N., Bearss K., Scahill L. (2018). Exploring sleep quality of young children with autism spectrum disorder and disruptive behaviors. Sleep Med..

[31] Tonge B., Einfeld S., Krupinski J., Mackenzie A., McLaughlin M., Florio T. (1996). The use of factor analysis for ascertaining patterns of psychopathology in children with intellectual disability. J. Intellect. Disabil. Res..

[32] Abidin R.R. (2012). Parenting Stress Index, Fourth Edition Professional Manual.

[33] Dardas L.A., Ahmad M. (2014). Psychometric properties of the Parenting Stress Index with parents of children with autistic disorder. J. Intellect. Disabil. Res..

[34] Valicenti-McDermott M., Lawson K., Hottinger K., Seijo R., Schechtman M., Shulman L., Shinnar S. (2015). Parental stress in families of children with autism and other developmental disabilities. J. Child Neurol..

[35] Kessler R.C., Andrews G., Colpe L.J., Hiripi E., Mroczek D.K., Normand S.-L., Walters E.E., Zaslavsky A.M. (2002). Short screening scales to monitor population prevalences and trends in non-specific psychological distress. Psychol. Med..

[36] Keenan B.M., Newman L.K., Gray K.M., Rinehart N.J. (2016). Parents of children with ASD experience more psychological distress, parenting stress, and attachment-related anxiety. J. Autism Dev. Disord..

[37] Rutter M., Bailey A., Lord C. (2003). Social Communication Questionnaire.

[38] Hertzog M.A. (2008). Considerations in determining sample size for pilot studies. Res. Nurs. Health.

[39] Cohen J. (1992). A power primer. Psychol. Bull..

[40] Ferguson C.J. (2009). Is psychological research really as good as medical research? Effect size comparisons between psychology and medicine. Rev. Gen. Psychol..

[41] StataCorp (2017). Stata Statistical Software: Release 15.

[42] Adams H.L., Matson J.L., Cervantes P., Goldin R.L. (2014). The relationship between autism symptom severity and sleep problems: Should bidirectionality be considered?. Res. Autism Spectr. Disord..

[43] Tudor M.E., Hoffman C.D., Sweeney D.P. (2012). Children with autism: Sleep problems and symptom severity. Focus Autism Other Dev. Disabil..

[44] Howlin P. (2000). Autism and intellectual disability: Diagnostic and treatment issues. J. R. Soc. Med..

[45] Mazurek M.O., Petroski G.F. (2015). Sleep problems in children with autism spectrum disorder: Examining the contributions of sensory over-responsivity and anxiety. Sleep Med..

[46] Tsermentseli S., Kouklari E.-C. (2021). Impact of child factors on parenting stress of mothers of children with autism spectrum disorder and intellectual disability: A UK school-based study. Early Child Dev. Care.

[47] Hayes S.A., Watson S.L. (2013). The impact of parenting stress: A meta-analysis of studies comparing the experience of parenting stress in parents of children with and without autism spectrum disorder. J. Autism Dev. Disord..

[48] Miranda A., Mira A., Berenguer C., Rosello B., Baixauli I. (2019). Parenting stress in mothers of children with autism without intellectual disability. Mediation of behavioral problems and coping strategies. Front. Psychol..

[49] Peters-Scheffer N., Didden R., Korzilius H. (2012). Maternal stress predicted by characteristics of children with autism spectrum disorder and intellectual disability. Res. Autism Spectr. Disord..

[50] Tilford J.M., Payakachat N., Kuhlthau K.A., Pyne J.M., Kovacs E., Bellando J., Williams D.K., Brouwer W.B.F., Frye R.E. (2015). Treatment for sleep problems in children with autism and caregiver spillover effects. J. Autism Dev. Disord..

[51] Hodge D., Hoffman C.D., Sweeney D.P., Riggs M.L. (2013). Relationship between children’s sleep and mental health in mothers of children with and without autism. J. Autism Dev. Disord..

[52] Díaz-Román A., Zhang J., Delorme R., Beggiato A., Cortese S. (2018). Sleep in youth with autism spectrum disorders: Systematic review and meta-analysis of subjective and objective studies. Evid. Based Ment. Health.

[53] Gringras P., Gamble C., Jones A.P., Wiggs L., Williamson P.R., Sutcliffe A., Montgomery P., Whitehouse W.P., Choonara I., Allport T. (2012). Melatonin for sleep problems in children with neurodevelopmental disorders: Randomised double masked placebo controlled trial. BMJ.

[54] Papadopoulos N., Sciberras E., Hiscock H., Mulraney M., McGillivray J., Rinehart N. (2015). The efficacy of a brief behavioral sleep intervention in school-aged children with ADHD and comorbid autism spectrum disorder. J. Atten. Disord..

[55] Malow B.A., Adkins K.W., Reynolds A., Weiss S.K., Loh A., Fawkes D., Katz T., Goldman S.E., Madduri N., Hundley R. (2014). Parent-based sleep education for children with autism spectrum disorders. J. Autism Dev. Disord..

